# Multicellular Interactions in 3D Engineered Myocardial Tissue

**DOI:** 10.3389/fcvm.2018.00147

**Published:** 2018-10-23

**Authors:** Maedeh Zamani, Esra Karaca, Ngan F. Huang

**Affiliations:** ^1^School of Medicine, The Stanford Cardiovascular Institute, Stanford University, Stanford, CA, United States; ^2^Department of Cardiothoracic Surgery, Stanford University, Stanford, CA, United States; ^3^Veterans Affairs Palo Alto Health Care System, Palo Alto, CA, United States

**Keywords:** engineered myocardium, cardiovascular tissue engineering, co-culture, cardiomyocyte, endothelial cell, fibroblast, stem cell

## Abstract

Cardiovascular disease is a leading cause of death in the US and many countries worldwide. Current cell-based clinical trials to restore cardiomyocyte (CM) health by local delivery of cells have shown only moderate benefit in improving cardiac pumping capacity. CMs have highly organized physiological structure and interact dynamically with non-CM populations, including endothelial cells and fibroblasts. Within engineered myocardial tissue, non-CM populations play an important role in CM survival and function, in part by secreting paracrine factors and cell-cell interactions. In this review, we summarize the progress of engineering myocardial tissue with pre-formed physiological multicellular organization, and present the challenges toward clinical translation.

## Introduction

**Figure 1:**
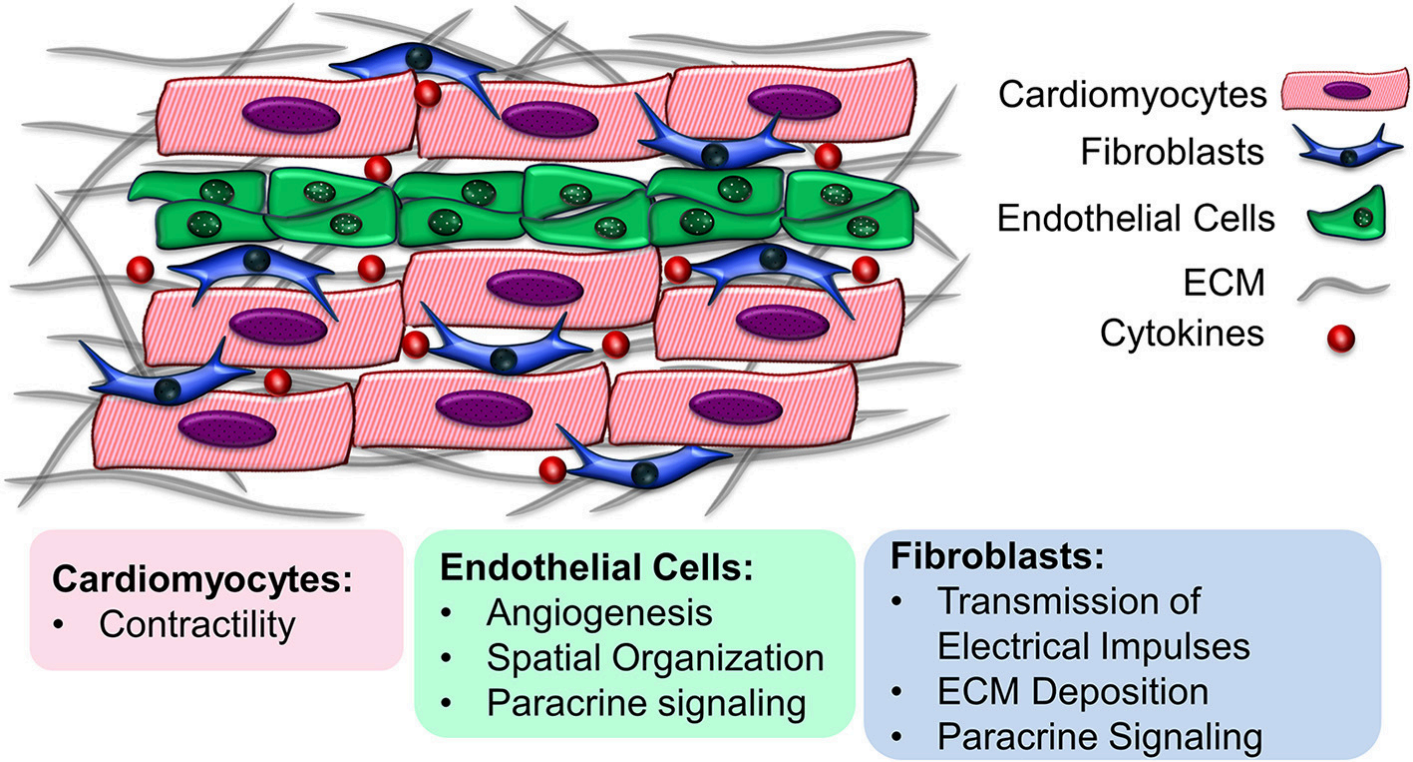
Multicellularity of the myocardial environment. Cardiomyocytes interact with support cells such as endothelial cells and fibroblasts to maintain their cell function. The underlying extracellular matrix also provides instructive cues that regulate cell survival and function.

The myocardium is a highly organized complex organ with multicellular structure. Besides the contractile cardiomyocytes (CMs), other important non-myocyte (non-CM) populations include endothelial cells (ECs), fibroblasts (FBs), vascular smooth muscle cells (SMCs), neuronal cells, and immune cells (1). In general, myocardial tissue is composed of 30–40% CMs and 60–70% non-CMs, although the cell population ratio varies regionally in the myocardium and across various species, as well as during cardiac development (2–5). Each cellular component has a distinct role in cardiac function. CMs mainly contribute to the conduction of electrical impulses and contraction, while non-CMs are responsible for vascularization, secretion of extracellular matrix (ECM) components and responding to myocardial injuries caused by oxygen depletion during ischemic heart diseases such as myocardial infarction (2). Apart from their individual functions, CMs and non-CMs interact with one another to regulate cellular organization, differentiation, viability and function. By mediating the production and transmission of the electrical, biochemical and mechanical signals in the cardiac microenvironment, non-CMs can regulate the function of CMs (2). The intercommunication among CMs and non-CMs is mediated through cell-cell interactions, soluble signaling proteins secreted by neighboring cells known as paracrine factors, and ECM-mediated crosstalk (6). Understanding the multicellular interactions in myocardial tissue is critically important, not only to provide insight into healthy and diseased cardiac microenvironments, but also to enable the development of cardio-mimetic tissue culture systems for cardiovascular regeneration applications. This review presents the recent advances in the development of functional cardiac tissue, with emphasis on the heterocellularity of the engineered constructs, toward resembling the multicellular structure of the myocardial environment (Figure 1). The importance of the cellular components as well as the physicochemical properties of the ECM in recreating native heart-like tissue are explored.

## *In vitro* Co-culture systems

Recent *in vitro* studies on generation of implantable cardio-mimetic units have shown great potential for cardiac regeneration, by developing engineered constructs with cellular, extracellular and biomolecular composition resembling native heart tissue. With respect to the cellular composition, various CM and non-CM cell types with different origin, age and activation/differentiation state have been employed for developing cardio-mimetic tissue constructs (7). Due to difficulties associated with long-term maintenance of adult CMs *in vitro*, neonatal CMs, embryonic stem cell-derived CMs (ESC-CMs) and induced pluripotent stem cell-derived CMs (iPSC-CMs) have been extensively explored in cardiac regeneration (8, 9). The use of these less differentiated cell sources signifies the importance of developing a multicellular microenvironment *in vitro*, in which the interactions with non-CM cells can improve the maturity of CMs in the culture system. Here we describe the progress in recreating the cardiac heterocellular microenvironment using CM-EC and CM-FB co-culture systems, and the importance of non-CMs in recapitulating a more biologically relevant microenvironment. We also present an overview of stem cell-based approaches toward developing multicellular tissue constructs, using stem cell-derived CMs/ECs/FBs. Although other cell types like neural cells and immune cells are important in controlling the CM contractility and hypertrophy, these cell types are discussed in greater detail elsewhere (10, 11).

### Fibroblasts (FBs)

FBs comprise the major non-CM cellular composition in the adult heart, where each CM is bordered by at least one FB (5, 12, 13). FBs play an essential role in maintaining myocardial structure and mediating CM function by depositing ECM components (6, 14), secreting soluble paracrine factors such as platelet-derived growth factor-β (PDGF-β) and basic fibroblast growth factor (bFGF), and propagating electrical signaling through gap junction proteins (e.g., connexin-43), in cell-cell contacts (15, 16). FBs can transmit electrical impulses between CMs with over 100 μm distance through intercellular gap junctions, contributing in synchronized CM contractions (15, 17, 18). Under the ischemic conditions, the inflammatory mediators such as transforming growth factor-β may induce ECM synthesis by FBs, resulting in cardiac fibrosis (11).

It has been demonstrated that CM-FB co-culture can preserve the polarized morphology of CMs and induce the long-term expression of CM gap junction protein connexin-43 that is responsible for CM-FB heterocellular coupling, resulting in long-term synchronized contraction of engineered cardiac tissue (17). There is evidence that FBs can also modulate the biological properties of CMs through the secretion of various paracrine factors, without direct cell-cell contact. For example, secretion of periostin by FBs has been shown to induce the proliferation of CMs both *in vitro* and *in vivo* (19). Soluble vascular cell adhesion molecule-1 (VCAM-1) secreted by cardiac FBs induced proliferation of mouse ESC-CMs, resulting in higher number of contractile cells and better propagation of extracellular electrical impulses (20). In addition to cell-cell contact signaling and paracrine factoring, the ECM components (e.g., fibronectin, elastin, and glycoproteins) and matrix metalloproteinases (MMPs) produced by FBs also work interdependently to regulate ECM homeostasis *in vivo*, with a determining role in cardiac remodeling and scar tissue formation after myocardial infarction (21, 22). Although CM-FB co-culture can improve CMs contractility and function, the abundance of FBs needs to be optimized, since increasing the FB population may impair CM maturation and reduce cardiac conduction speed, action potential propagation and membrane resting potential by the secretion of paracrine factors (23, 24).

### Endothelial cells (ECs)

As a highly metabolic cell type, CMs reside within 2–3 μm distance from nearest capillary (25, 26). CMs require ECs in the capillary network to provide oxygen and nutrients. ECs have a pivotal role in protecting CMs against ischemic injuries by secreting neuregulin and PDGF-β and reducing fibrotic tissue formation after myocardial infarction (27, 28). An important goal of CM-EC co-culture is to create a vascularized cardio-mimetic tissue. ECs have also shown to be a dynamic mediator of CM spatial organization, contraction, survival and function in a complex heterocellular physiological platform (26, 29–31). ECs in co-culture can promote neovascularization by forming cellular networks within the engineered tissue unit and producing angiogenic cytokines such as vascular endothelial growth factor (VEGF), basic fibroblast growth factor (bFGF), and hepatocyte growth factor (HGF), which can eventually enhance the capillary density of the implanted endothelialized cardiac tissue *in vivo* (27). ECs can also interact with CMs through autocrine and paracrine signaling factors, such as neuregulin and PDGF-β to enhance CM survival (28, 29, 31), and nitric oxide and endothelin-1 to improve CM contractility (26, 29, 32). Besides paracrine signaling, ECs direct spatial organization of the CMs through cell-cell contact. CMs formed interconnected organized structures along capillary-like network in the co-culture system, but this organization was not observed when CMs were cultured with EC-conditioned medium (29).

### Pluripotent stem cell-derived CMs/FBs/ECs

Recent advances in efficient differentiation of pluripotent stem cells to cardiac cells represented a new unlimited source of cells for developing cardiac tissues, particularly due to the limited availability of donor primary CMs. However, the survival rate, maturation, and function of these less differentiated cells are highly dependent on the biochemical, mechanical and topographical properties of their microenvironment. The co-culture of both embryonic and induced pluripotent stem cells-derived CMs (ESC-CMs and iPSC-CMs) with non-CMs of different sources demonstrated to preserve the CMs phenotype and function through cell-cell contact and biochemical cues (33–35). The scaffold-free patches created from human ESC-CMs, ESC-ECs and fibroblasts exhibited electrically-paced contraction and more myocardially-relevant passive mechanical properties, compared to ESC-CMs in monoculture. Transplantation of multi-culture pre-vascularized tissue constructs showed high engraftment and anastomosis to the animal's coronary artery, unlike the patches consisted of pure ESC-CMs that did not survive after implantation (36). Entirely iPSC-derived cardiac patches composed of iPSC-CMs and iPSC-ECs were developed using aligned nanofibrous scaffolds. The multicellular cardiac patches demonstrated improved maturity of iPSC-CMs, as indicated by increased sarcomeric length and myosin heavy chain gene expression that mimicked mature adult CMs (33). Entirely autologous iPSC-derived multicellular cardiac tissues can potentially address the problems associated with allogeneic cardiac tissues for future clinical applications. Although multicellular interactions are critical for promoting pluripotent stem cell survival and function, the ratio of the non-CMs need to be optimized in the tissue construct. A high fraction of non-CMs may adversely affect iPSC-CMs electrical conduction and maturation through the production of various ECM proteins, i.e., collagen, fibronectin, laminin (33, 34).

## Engineering functional co-cultured tissues

The formation of a functional cardiac construct comprising multi-cellular components requires the cells to be exposed to an *in vitro* environment that can efficiently support their proliferation, differentiation, organization, and function. Taking advantage of advances in the field of tissue engineering, multi-cellular cardiac constructs have been fabricated over the years using various cell culture techniques and scaffolding materials, to optimize the regenerative ability of the engineered tissue. In general, engineering a biomimetic cardiac tissue *in vitro* aims at generating a highly aligned, contractile, and vascularized cardiac construct in a three-dimensional (3D) microenvironment. In this section, we present some of the main techniques developed for fabrication of 3D co-cultured cardiac constructs to enhance their cellular organization and function.

### Cell sheet engineering

Cell sheet engineering, the technique for constructing 3D functional tissues by layering two-dimensional (2D) cell sheets harvested from thermo-responsive cell culture surfaces, is an efficient approach for developing layered CM sheets co-cultured with non-CMs. A 3D cardiac tissue made through cell sheet technology may resemble dense cellular population and tight interconnection with gap junctions, which facilitates the exchange of biomolecules and ions resulting in electrically synchronized contraction. Synchronized contractions was observed for the layered CMs, indicating the electrical communication between layers of CMs in 3D construct (37). However, sufficient oxygen and nutrient diffusion into the layered CMs sheets is critically important, particularly due to the dense cell density in 3D constructs (38). Multicellular 3D platforms were also developed either by layering sheets of heterogeneous cells (27) or mono-cultured layers of different cell types (39). When EC-CM heterogeneous sheets were layered to develop pre-vascularized 3D constructs, it significantly enhanced capillary density in the graft, as compared with mono-cultured cell sheets *in vivo* (27). Alternating sheets of neonatal CMs and FBs could also form gap junctions between heterogeneous layers, resulting in synchronized beating of the 3D tissue (39).

### Polymer scaffolds

Polymer porous scaffolds with a wide range of biochemical, mechanical and topographical properties have been developed for creating cardiac tissues, with the aim of providing a temporary ECM-like support to guide the cells growth, organization and function. The native ECM in the heart is a highly anisotropic (parallel-aligned), elastic and fibrillar network, and significant effort has been made to recapitulate this structure (40, 41). Polymer scaffolds can mediate the cross-talk between the cells in co-culture through its chemical, mechanical and electrical properties, as well as the architectural design of the scaffold which determines the cellular spatial patterning, cell-cell spacing, and the transmission of biomolecular/electrical signals. One advantage of polymer porous scaffolds over cell sheet technology is the ease of engineering 3D constructs. Nanofibrous scaffolds possessing fibrous ECM-like architecture with interconnected pores have been widely explored for generating cardiac tissues (17, 40, 42, 43). The CM-FB and CM-EC co-culture systems on chitosan nanofibers resulted in polarized CMs morphology and maintenance of long-term function (17). Conductive nanofibrous yarns containing carbon nanotubes were incorporated in GelMA hydrogel to produce 3D composite scaffolds mimicking native cardiac ECM. The co-culture of CMs and ECs on the nanofibers yarn and hydrogel construct respectively resulted in aligned CMs morphology in a uniform ECs network, capable of developing pre-vascularized tissue for myocardial regeneration (44). 3D porous scaffolds were also developed through other techniques such as salt leaching (35). Poly(l-lactic acid)/poly(lactide-co-glycolide (PLLA/PLGA) porous sponges seeded with a tri-culture system (i.e., ESC-derived CMs/FBs/ECs) showed higher density of functional vasculature integrated with host myocardial microvasculature, when compared to sponges consisting of mono-cultures of ESC-CMs (35).

Since CMs have highly organized physiological structure for driving efficient electromechanical coupling and contractility, much attention has been placed to recreate endogenous CM spatial patterning using instructive polymeric biomaterials. Spatial patterning of CMs on electrospun fibrous scaffolds (45) with aligned organization led to CM alignment along the direction of the fibers, increased contractile strength, and synchronized beating, compared to cells grown on the randomly oriented scaffolds, owing to improved intercellular crosstalk and exchange of ions via gap junctions (46). CM function was further improved when the aligned scaffolds were co-cultured with FBs or ECs. Similar effects of spatial patterning and improved contractile strength has also been reported using iPSC-CMs (33). It was also demonstrated that the alignment of fibrous scaffolds made of elastomeric polyurethane can improve the parallel organization of ESC-CMs and sarcomeric length in a CM-FB co-culture system (42). The roles of cell-cell contact and paracrine factors were further examined when anisotropic scaffolds seeded with rat CMs were co-cultured with scaffolds seeded with fibroblasts with either direct contact or indirect contact (paracrine interaction only). Interestingly, regardless of direct or indirect contact, the presence of FBs improved CM phenotype and aligned sarcomere organization, suggesting that both spatial patterning and paracrine factors are important for CM function (43). Together, these studies suggest that polymer scaffolds and anisotropic spatial patterning promote heterocellular cultures for improved CM function.

### Hydrogels

Hydrogel systems mainly derived from natural sources such as Matrigel (47), fibrin (7), collagen I (48), gelatin methacrylate (GelMA) (49), and peptide hydrogel (29), have been used as substrates to prepare 3D heterocellular units for cardiac regeneration. To develop hydrogel-based 3D constructs, a solution of gelling material mixed with cellular components is usually casted into a mold where the gelation process occurs. Although the mechanical properties of hydrogel systems are lower compared to polymer scaffolds, they are normally more compliant than polymers (23). Cellular spreading, the ability to deform the hydrogel, and nutrient exchange are important factors in obtaining a functional contractile tissue construct (50). Studies show that, unlike ESC-CMs cultured in a fibrin-based hydrogel, ESC-CM/FB co-cultures could successfully compact and remodel the hydrogel substrate to generate macroscopically synchronized action potential propagation (23). It was also demonstrated that geometrical properties of the micropatterned hydrogel platform can influence the synchronized contraction of neonatal CMs-FBs co-culture in a GelMa hydrogel (49, 51). The authors found that the presence of FBs led to elongation of CMs, increased integrin expression levels, and increased connexin43 gap junctions, compared to CM-only cultures.

## Emerging technologies

In recent years multicellular systems design has been improved with the emerging bioprinting and organ-on-a-chip (OOC) technologies that provide new approaches for basic research and drug discovery. Since the geometry and spatial arrangement of different cell types are important for their function, 3D bioprinting is a useful technology for turning digital designs into precisely organized 3D cultures. Bioprinting uses ECM proteins such as collagen, gelatin, hyaluronic acid, alginate, and de-cellularized ECMs as bioinks (52) to construct digitally designed 3D cultures. To overcome the problem of degradation of scaffolds *in vivo*, bioprinting has also been used to create cardiac tissue consisting of multicellular aggregates and omitting biomaterials altogether (53). Ong et al. created cardiac patches using spheroids of CMs, ECs and FBs that were 3D printed on a needle array and allowed to fuse. They showed that the resulting patch was electrically active and upon implantation *in vivo* resulted in vascularization and engraftment (54).

Recent research on organoid cultures showed that stem cells have the intrinsic capacity to self-assemble and differentiate into 3D tissues that can replicate some of the properties of the native organ, which suggests that it may not be necessary to 3D print the entire tissue. The cardiac organoids developed so far remain in the more immature stages and resemble fetal heart (55). Bioprinting can be employed to guide the cardiac organoid formation by creating patterns and guidance cues in its microenvironment (56).

Some of the limitations of traditional static 3D cultures such as lack of geometric organization, absence of a clear tissue interface, and the accumulation of secreted compounds into spent media can be overcome in a microfluidic platform. Microfluidic platforms enable tissues of different cell types to be separated from one another by porous membranes to allow for molecular crosstalk. These OOC platforms allow for longer term cultures, owing to the circulating factors, nutrients and oxygen in the microfluidic system. The circulation of media in the capillaries give rise to mechanical factors such as fluid shear stress and allows for the monitoring of secreted factors and metabolites in real-time. The microfluidic chips also make it possible to investigate changes in the morphology and migration of cells (57). This approach has been used to create myocardial OOCs using CMs and ECs derived from human iPSCs to create models for drug screening and disease modeling (58, 59).

## Limitations and future directions

*In vitro* co-culture systems play a significant role in advances in cardiac tissue engineering over the last years. Using heterocellular engineered cardiac tissues, we are closer to developing functional vascularized tissues by taking advantage of complex and dynamic crosstalk between various types of cells and their interactions with ECM. However, extensive studies are yet required to fully understand the distinct roles of each individual cellular component and their interactions, to optimize engineered vascularized cardiac tissue. The *in vitro* co-culture systems are capable of creating physiologically relevant cardiac tissues for regenerative purposes. However, the read-out of multicellular systems is highly complexed and more innovative cell culture systems and characterization techniques are still required to precisely understand the heterocellular communication at a single-cell level to progress in the field. Although 3D scaffolds may better recapitulate the native heart ECM network, the difficulties associated with oxygen and nutrient diffusion, cell infiltration and viability, vascularization, and degree of scaffold remodeling may impair the clinical translation of multicellular constructs made by using 3D scaffolds. The source of the cells as well as the age, differentiation state and cell ratios of the co-culture platforms are important considerations influencing the functions of engineered vascularized cardiac tissues. Despite advances in employing ESC-CMs and iPSC-CMs for cardiac regeneration, it is critical to design cellular microenvironment to maintain characteristics of these differentiated cells by introducing relevant electrical, molecular, mechanical and topographical cues in long-term culture systems. The use of advanced materials such as conductive scaffolds may also facilitate the electrophysiological communication among the cells of multicellular environment. As more advanced techniques emerge in the field of tissue engineering, more biologically relevant native myocardial-like tissues will be developed for regenerative applications.

## Author contributions

MZ and NH conceived the content of the manuscript. MZ and EK analyzed the literature and wrote the manuscript. NH critically reviewed and revised the manuscript. All authors read and approved the submitted version.

### Conflict of interest statement

The authors declare that the research was conducted in the absence of any commercial or financial relationships that could be construed as a potential conflict of interest.
